# Dietary insulin index and dietary insulin load in relation to non-alcoholic fatty liver disease: a cross-sectional study

**DOI:** 10.1017/S1368980024001149

**Published:** 2024-09-26

**Authors:** Amir Motamedi, Shahab Alizadeh, Saeed Osati, Tahereh Raeisi, Reza Homayounfar

**Affiliations:** 1 Student Research Committee, Shiraz University of Medical Sciences, Shiraz, Iran; 2 Development and clinical research center, Baharloo Hospital, Tehran University of Medical Sciences, Tehran, Iran; 3 Department of Clinical Nutrition, School of Nutritional Sciences and Dietetics, Tehran University of Medical Sciences (TUMS), Tehran, Iran; 4 Nutritional Health Research Center, Lorestan University of Medical Sciences, Khorramabad, Iran; 5 National Nutrition and Food Technology Research Institute, Faculty of Nutrition Sciences and Food Technology, Shahid Beheshti University of Medical Sciences, Tehran, Iran; 6 Department of Medicine, Hormozgan University of Medical Sciences, Bandar Abbas, Iran

**Keywords:** Fatty liver disease, Dietary insulin index, Dietary insulin load

## Abstract

**Objective:**

Postprandial hyperinsulinaemia plays a key role in the development of non-alcoholic fatty liver disease (NAFLD). Diet is a potential factor affecting serum insulin levels. This study aimed to examine the relations of dietary insulin index (DII) and dietary insulin load (DIL) to the risk of NAFLD.

**Design:**

This study was a cross-sectional study. DII and DIL were calculated using the dietary data obtained from the FFQ. Fatty liver index ≥ 60 and the confirmation of a gastroenterologist were required to diagnose NAFLD.

**Setting:**

Community-based study.

**Participants:**

A total of 3158 people (46·7 % male), aged 40·57 ± 8·25 years, participated in this study in Tehran, Iran from April 2016 to December 2019.

**Results:**

The prevalence of NAFLD was 29·9 % (21·59 % in males and 33·74 % in females). In the fully adjusted model controlled for sex, age, energy intake, BMI, smoking, physical activity and education, DII was significantly associated with the increased risk of NAFLD in males (OR: 2·74, 95 % CI = 1·75, 4·31; *P*-trend = ≤0·001) and females (OR: 2·26, 95 % CI = 1·39, 3·69; *P*-trend = 0·005). A significant relationship was also detected between DIL and NAFLD in females (OR: 2·90, 95 % CI = 1·70, 4·93; *P*-trend ≤0·001) but not in males (OR: 1·33, 95 % CI = 0·84, 2·10; *P*-trend = 0·13).

**Conclusions:**

Adherence to a diet with a high DII and DIL may be related to the increased risk of NAFLD. These results may be useful for healthcare providers to design appropriate preventive measures for people at risk of NAFLD.

Nonalcoholic fatty liver disease (NAFLD), defined as fat accumulation in the liver in people without considerable alcohol intake, is the leading liver complication globally and is one of the main reasons for liver transplant, cirrhosis and hepatocellular carcinoma^(1)^. The prevalence of NAFLD is growing globally, with an incidence of about 25 % and 30 % in Asian and Western countries, respectively^(2,3)^. NAFLD is toughly linked to obesity, metabolic syndrome, insulin resistance, type 2 diabetes mellitus, hypertension and increased risk of mortality^(4)^. Thus, it is critical to recognise its risk factors and develop effective preventive approaches against NAFLD.

Postprandial hyperglycaemia and hyperinsulinaemia play key roles in the development of NAFLD^(5,6)^. Among dietary components, carbohydrates have a central role in postprandial hyperglycaemia and thus are the chief stimulus for insulin secretion^(7)^. It has been identified that a diet with a high dietary glycaemic index (GI) and glycaemic load (GL), as indicators of carbohydrate quality and quantity, may be associated with an increased risk of NAFLD^(8)^. Nevertheless, postprandial insulin concentrations are not always proportionate to blood glucose levels, and other dietary factors, including some amino acids, fructose and certain fatty acids can influence the insulinogenic impacts of foods^(9)^. Certain foods with high amounts of fat and protein might induce a significant insulin release, despite a minor increase in glucose concentrations^(10)^. Therefore, GI and GL have inadequate capacity for precise evaluation of insulin responses of all dietary components^(11)^. Recently, dietary insulin index (DII) and dietary insulin load (DIL) have been developed for this purpose. The DII measures the amount of postprandial insulin secretion after consumption of each food in comparison with an isoenergetic portion of a reference food (white bread or glucose). DIL is calculated by multiplying the DII of each food by its energy content and the consumption frequency^(12)^. Unlike glycaemic scores, insulin indices are calculated according to the calorie contents of food items and directly evaluate the postprandial insulin secretion in response to the composite meals irrespective of the macronutrient compositions of diet, even for carbohydrate-free food items, providing an accurate tool to explore diet–disease relationships because insulin index is directly based on insulin response^(13)^.

Epidemiological studies have revealed that adherence to a diet with a high DII and DIL is related to metabolic disorders, such as metabolic syndrome^(7)^, insulin resistance^(12)^, hyperlipidaemia^(14)^, obesity^(15)^ and inflammation^(16)^. Despite this, evidence regarding the relations of DII and DIL to NAFLD is very rare. Therefore, the present study aimed to explore the relations of DII and DIL to NAFLD among a large population of Iranian adults.

## Methods and materials

### Participants

A total of 3158 subjects, aged 40·57 ± 8·25 years, were included in this cross-sectional study. From April 2016 to December 2019, participants were randomly selected with the use of a convenience sampling approach among patients referred to a university-affiliated nutrition counselling centre in Tehran, Iran. The inclusion criteria were age ≥18 years and no history of considerable alcohol consumption (< 20 g/d for females and < 30 g/d for males). Individuals were excluded in case of the presence of diseases, such as diabetes mellitus, thyroid disorders, hepatic disease, malignancy and consumption of blood sugar/lipid lowering or chemotherapy drugs, which affect metabolic status. Individuals who used dietary supplements or followed a weight loss diet during the last 3 months were also excluded. A written informed consent was obtained from all participants. The study protocol was based on the Helsinki Declaration and was approved by the Ethics Committee of Fasa University of Medical Sciences (IR.FUMS.REC. 1396·230).

### Demographic, anthropometric and clinical measurements

Demographic data such as age, sex, education, disease history, as well as drug use and smoking were obtained using a self-administer questionnaire. The height of participants was determined using a stadiometer with a precision of 0·1 cm. Weight was measured by a digital scale with a precision of 0·1 kg (Seca 767, Japan). BMI was calculated as weight (kg)/square of height (m^2^). Waist circumference (WC) was assessed by measuring the midway area between the iliac crest and the lower edge of the lowest rib. Moreover, the level of physical activity was assessed using a validated version of the International Physical Activity Questionnaire and reported as the metabolic equivalent per day (MET/day)^(17)^.

For all participants, 10 ml of peripheral blood was obtained after 12 h of overnight fasting. The blood samples were centrifuged at 2500 rpm for 6–8 min to separate serum. Serum fasting blood glucose (FBS), fasting insulin, alanine aminotransferase (ALT), gamma-glutamyl transferase, aspartate aminotransferase, TAG, HDL and total cholesterol (TC) were assessed by an enzymatic approach with the use of the Pars Azmoon Commercial kits. The concentration of LDL was estimated using the Friedewald formula^(18)^. Homeostasis model assessment formula for insulin resistance (HOMA-IR: (FBS (mg/dl) × fasting insulin (mU/ml))/405) and homeostasis model assessment formula for *β*-cell function (HOMA-B%: (360 × fasting insulin (mU/ml))/FBS (mg/dl) - 63) were used to evaluate insulin resistance and beta cell activity, respectively^(19)^. After 15 min of rest, systolic blood pressure and diastolic blood pressure were assessed twice (sphygmomanometer, mercury, ALPK1, Japan), with a 15-minute interval, and the mean of the two measurements was recorded as the blood pressure of the subjects.

The diagnosis of NAFLD was based on the fatty liver index (FLI), as described previously^(20)^ using the following formula:

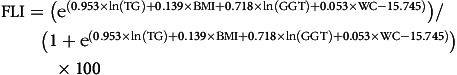




Then, the confirmation of a gastroenterologist using a fibroscan was required to consider patients as NAFLD. The FLI score of lower than < 30 was defined as a normal liver, the scores of 30–59 were considered as having intermediate FLI and the score of ≥ 60 was considered as the cut-off point of having NAFLD^(20)^.

### Dietary assessment and the calculation of DIL and DII

The common food intake of subjects over the previous year was evaluated with the use of a validated 168-item FFQ^(21)^. An expert dietitian completed FFQ and asked the participants to report the frequency of intake (daily, weekly or monthly) and amount of intake according to standard portion sizes of food items based on the household measures usually used by Iranians through a face-to-face interview. Then, portion sizes of food items were converted to grams/day. The daily intakes of energy and nutrients were computed with the use of the United States Department of Agriculture database using the Nutritionist IV software modified for the Iranian food. The test procedure of DII is provided by the study of Brand-Miller *et al.* ^(10)^. Briefly, DII is defined as the incremental insulin AUC over 2 h following the ingestion of a 1000-kJ portion of the food item divided by the AUC after intake of a 1000-kJ portion of the reference food (glucose). In the present study, DII for fifty-nine food items available in the FFQ (see online supplementary material, Supplemental Table S1) was obtained based on the DII from studies by Sadeghi *et al.* ^(7)^, Bao *et al.* ^(22)^ and Bell *et al.* ^(23)^. To compute DII of the diet, we first calculated DIL of the diet. The insulin load of each food item was computed by multiplying the insulin index of that food item by energy content and the frequency of the consumption of that food item: (insulin index of that food × kilocalories per serving × servings per day). Then, insulin loads of all food items were summed up to obtain the DIL of the whole diet. Each unit of DIL represents the equivalent insulin response generated by 1 kcal of glucose. The DII for the whole diet, which is the weighted mean of insulin index values for each of the component foods, was computed as DIL/total energy intake^(7,10)^.

### Statistical analysis

The normality of data was measured using the Kolmogorov–Smirnov test and data followed a normal distribution. The participants were categorised according to the DII and DIL quartiles. The one-way ANOVA and *I* ^2^ tests were applied to examine the difference in quantitative and qualitative variables among the DII and DIL quartiles, respectively. Data were presented as mean ± sd or frequency (percent) for quantitative and categorical variables, respectively. The binary logistic regression analysis was used to calculate the OR and 95 % CI for the links of DII and DIL to the risk of NAFLD according to the three models. Model 1 was a crude model without an adjustment for the covariates; model 2 was controlled for sex, dietary energy intake and age; model 3 included covariates adjusted for in model 2 plus smoking status, level of education and physical activity and model 4 included covariates adjusted for in model 3 plus BMI. All statistical tests were carried out with the use of SPSS (version 23) and *P* values <0·05 were considered as statistically significant.

## Results

Among 3158 people (46·7 % male) participating in this study, a total of 943 (29·9 %) individuals (21·59 % in males and 33·74 % in females) were diagnosed to have NAFLD. The scores of DII and DIL ranged from 35·93 to 126·92 and 372·02 to 1302·15, respectively. The mean ages of NAFLD patients and healthy subjects were 42·26 ± 8·19 and 39·86 ± 8·17, respectively. The mean BMI was 27·35 ± 3·85 in healthy individuals and 28·14 ± 3·96 in NAFLD patients. The mean DII and DIL were 78·21 ± 19·75 and 784·06 ± 192·35, respectively. The basic characteristics of participants across quartiles of DII and DIL are presented in Tables 1 and 2, respectively. Subjects in the higher quartiles of DII and DIL had a higher prevalence for NAFLD and had higher age, energy intake, weight, height, BMI, waist circumference, FBS, fasting insulin, HOMA-IR, HOMA-B, LDL, TAG, TC, systolic blood pressure, diastolic blood pressure and liver enzymes (ALT, AST, GGT) but lower levels of physical activity, HDL and frequency of females (*P* < 0·05). Subjects with higher scores for DII and DIL had significantly higher intakes of energy and refined grains but consumed lower amounts of whole grains, legumes, processed meats, nuts, dairies, vegetables, fruits and red meat (*P* < 0·001) (see online supplementary material, Supplemental Table S2). Among different food groups, based on the Pearson’s correlation coefficient, refined grains had the highest positive correlation with DII (*r* = 0·51, *P* < 0·001) and DIL (*r* = 0·26, *P* < 0·001), while fruits and dairies had the highest negative correlation with DII (*r* = –0·52, P < 0·001) and DIL (*r* = –0·23, *P* < 0·001) (see online supplementary material, Supplemental Table S3).

**Table 1:** Demographic characteristics of participants across quartiles of dietary insulin index

Variables	Quartiles of dietary insulin index								
	Q1 (*n* 789) Range (35·93–61·73)		Q2 (*n* 790) Range (61·73–76·56)		Q3 (*n* 790) Range (76·57–93·21)		Q4 (*n* 789) Range (93·27–126·92)		*P*-value^*^
	Mean or *n*	sd or %	Mean or *n*	sd or %	Mean or *n*	sd or %	Mean or *n*	sd or %	
Dietary insulin index	53·90	5·56	69·05	4·31	85·07	4·74	104·81	7·70	<0·001
Dietary insulin load	582·27	95·65	714·33	119·77	843·17	121·43	996·48	124·99	<0·001
Female (n (%))	552	70·0	419	53·0	429	54·3	282	35·7	<0·001
NAFLD, yes (n (%))	117	14·8	208	26·3	261	33·0	357	45·2	<0·001
Smoker, yes (n (%))	92	11·7	107	13·5	127	16·1	126	15·9	0·10
Education, diploma and higher (n (%))	638	80·9	640	81·0	651	82·4	615	77·9	0·15
Age (year)	34·70	5·89	38·10	7·44	42·82	7·73	46·68	6·27	<0·001
Energy intake (kcal/d)	2314·37	368·98	2357·77	462·64	2486·25	476·41	2582·85	423·74	<0·001
Weight (kg)	80·91	6·88	84·21	9·11	90·80	8·66	95·78	6·97	<0·001
Height (cm)	161·43	12·57	162·91	13·16	162·14	13·07	163·84	13·54	0·002
BMI (kg/m^2^)	25·11	2·49	26·79	3·92	28·60	4·06	29·84	3·16	<0·001
Waist circumference (cm)	92·67	8·16	101·06	11·26	111·73	11·20	118·98	8·82	<0·001
Physical activity (MET/day)	29·48	4·06	27·18	5·19	24·68	4·80	23·35	3·81	<0·001
FBS (mg/dl)	96·56	9·25	103·28	11·30	110·64	11·13	117·91	9·35	<0·001
Insulin (µU/ml)	8·02	1·53	9·64	2·15	11·64	1·95	13·36	1·76	<0·001
HOMA-IR	9·19	1·30	10·14	1·50	11·25	1·47	12·28	1·38	<0·001
HOMA-B	66·70	8·36	70·27	9·64	73·51	10·47	78·07	9·46	<0·001
LDL (mg/dl)	92·59	6·78	98·67	7·81	104·60	8·06	111·45	8·06	<0·001
HDL (mg/dl)	47·18	4·27	43·79	4·90	40·80	4·55	37·59	3·93	<0·001
TAG (mg/dl)	135·24	10·66	138·78	13·53	145·21	13·74	149·16	10·34	<0·001
TC (mg/dl)	180·11	12·49	190·21	15·38	199·73	16·11	211·11	14·36	<0·001
SBP (mmHg)	12·35	0·66	12·59	0·81	12·79	0·81	12·92	0·74	<0·001
DBP (mmHg)	8·10	0·59	8·34	0·84	8·56	0·91	8·79	0·84	<0·001
ALT (IU/l)	40·73	9·86	44·43	11·96	51·23	11·46	56·67	9·99	<0·001
AST (IU/l)	34·60	8·82	38·14	10·76	43·49	11·10	47·84	9·92	<0·001
GGT (IU/l)	22·94	5·19	27·37	6·89	32·21	6·92	37·85	6·99	<0·001

Data are presented as mean ± sd or frequency (percent).

* Obtained from the one-way ANOVA or Chi-squared tests, where appropriate.

NAFLD, non-alcoholic fatty liver disease; FBS, fasting blood glucose; HOMA-IR, homeostatic model assessment for insulin resistance; HOMA-B, homeostatic model assessment for β-cell function; LDL, low-density lipoprotein; HDL, high-density lipoprotein; TC, total cholesterol; SBP, systolic blood pressure; DBP, diastolic blood pressure; ALT, alanine transaminase; AST, aspartate transaminase; GGT, gamma-glutamyltransferase.

**Table 2:** Demographic characteristics of participants across quartiles of dietary insulin load

Variables	Quartiles of dietary insulin load								
	Q1 (*n* 789) Range (372·02–622·12)		Q2 (*n* 790) Range (622·24–775·16)		Q3 (*n* 790) Range (775·49–931·65)		Q4 (*n* 789) Range (931·70–1302·15)		*P*-value^*^
	Mean or *n*	sd or %	Mean or *n*	sd or %	Mean or *n*	sd or %	Mean or *n*	sd or %	
Dietary insulin load	545·63	53·42	697·26	44·52	853·17	44·74	1040·20	80·21	<0·001
Dietary insulin index	57·55	9·43	70·67	12·03	84·70	13·13	99·92	12·56	<0·001
Female (n (%))	543	68·8	437	55·3	425	53·8	277	35·1	<0·001
NAFLD, yes (n (%))	136	17·2	178	22·5	273	34·6	356	45·1	<0·001
Smoker, yes (n (%))	97	12·3	107	13·5	113	14·3	135	17·1	0·11
Education, diploma and higher (n (%))	638	80·9	660	83·5	640	81·0	606	76·8	0·008
Age (year)	34·02	5·63	38·61	7·39	42·63	7·41	47·04	6·18	<0·001
Energy intake (kcal/d)	2286·04	345·46	2375·18	439·66	2503·27	506·05	2576·72	426·45	<0·001
Weight (kg)	80·34	6·70	84·72	8·92	91·05	8·94	95·60	6·79	<0·001
Height (cm)	161·51	12·40	162·34	13·72	162·55	12·74	163·92	13·46	<0·001
BMI (kg/m^2^)	25·07	2·47	26·68	3·74	28·51	4·09	30·07	3·15	<0·001
Waist circumference (cm)	91·74	7·68	101·21	10·85	112·40	10·45	119·08	8·68	<0·001
Physical activity (MET/day)	29·83	3·94	26·95	4·98	24·65	4·94	23·27	3·68	<0·001
FBS (mg/dl)	95·78	8·66	103·00	10·96	111·87	10·85	117·72	9·27	<0·001
Insulin (µU/ml)	7·83	1·48	9·70	1·96	11·75	1·92	13·38	1·71	<0·001
HOMA-IR	9·11	1·22	10·19	1·44	11·22	1·51	12·34	1·34	<0·001
HOMA-B	65·91	8·14	70·28	9·80	73·75	10·00	78·61	9·10	<0·001
LDL (mg/dl)	92·09	6·01	98·50	7·89	105·36	7·88	111·35	7·93	<0·001
HDL (mg/dl)	47·41	4·14	43·83	4·80	40·64	4·46	37·47	3·77	<0·001
TAG (mg/dl)	134·92	10·52	138·54	13·75	145·11	13·12	149·83	10·26	<0·001
TC (mg/dl)	179·91	12·06	189·59	15·83	200·68	15·63	210·99	14·13	<0·001
SBP (mmHg)	12·13	0·62	12·59	0·86	12·81	0·79	12·92	0·74	<0·001
DBP (mmHg)	8·10	0·60	8·32	0·83	8·57	0·90	8·80	0·85	<0·001
ALT (IU/l)	39·87	9·59	44·90	11·62	51·29	11·25	57·00	10·08	<0·001
AST (IU/l)	34·00	8·76	38·41	10·65	43·60	10·72	48·07	10·00	<0·001
GGT (IU/l)	22·55	4·86	27·30	6·54	32·65	6·98	37·86	6·90	<0·001

Data are presented as mean ± sd or frequency (percent).

* Obtained from the one-way ANOVA or Chi-squared tests, where appropriate.

NAFLD, non-alcoholic fatty liver disease; FBS, fasting blood glucose; HOMA-IR, homeostatic model assessment for insulin resistance; HOMA-B, homeostatic model assessment for *β*-cell function; LDL, low-density lipoprotein; HDL, high-density lipoprotein; TC, total cholesterol; SBP, systolic blood pressure; DBP, diastolic blood pressure; ALT, alanine transaminase; AST, aspartate transaminase; GGT, gamma-glutamyltransferase.

Multivariable-adjusted odds ratios and corresponding 95 % CI for NAFLD among quartiles of DII and DIL for the total population, females and males are reported in Table 3. After controlling the analysis for the potential covariates, including sex, age, dietary energy intake, BMI, smoking status, physical activity and education levels, a significant direct relationship was found between DII (OR: 2·43, 95 % CI = 1·75, 3·37; *P*-trend = ≤0·001) and DIL (OR: 1·87, 95 % CI = 1·33, 2·63; *P*-trend ≤0·001) with the risk of NAFLD in people with the highest adherence, compared with those in the lowest quartile. In the subgroup analysis by sex, DII was a significant predictor for NAFLD in males (OR: 2·74, 95 % CI = 1·75, 4·31; *P*-trend = ≤0·001) and females (OR: 2·26, 95 % CI = 1·39, 3·69; *P*-trend = 0·005). A significant relationship was also detected between DIL and NAFLD for females (OR: 2·90, 95 % CI = 1·70, 4·93; *P*-trend ≤0·001). For males, the association of DIL with NAFLD disappeared in the fully adjusted model (OR: 1·33, 95 % CI = 0·84, 2·10; *P*-trend = 0·13), but it was significant when adjusted for age, sex and daily energy intake (OR: 1·75, 95 % CI = 1·18, 2·59; *P*-trend = 0·001) (Table 3).

**Table 3:** Logistic regression analysis for the association of dietary insulin index and dietary insulin load with the risk of NAFLD in the whole population (*N* 3158), males (*N* 1476) and females (*N* 1682)

			Model 1			Model 2			Model 3			Model 4		
			OR	95 % CI	*P*-value	OR	95 % CI	*P*-value	OR	95 % CI	*P*-value	OR	95 % CI	*P*-value
Total population	DII	Quartile 1	1			1			1			1		
		Quartile 2	2·05	1·59, 2·64	≤0·001	1·70	1·31, 2·20	≤0·001	1·60	1·23, 2·09	0·001	1·56	1·20, 2·04	0·001
		Quartile 3	2·83	2·21, 3·62	≤0·001	2·21	1·69, 2·90	≤0·001	2·00	1·50, 2·65	0·001	1·91	1·43, 2·56	≤0·001
		Quartile 4	4·74	3·72, 6·04	≤0·001	2·99	2·23, 4·02	≤0·001	2·57	1·88, 3·52	0·001	2·43	1·75, 3·37	≤0·001
		*P*-trend			≤0·001			≤0·001			0·001			≤0·001
	DIL	Quartile 1	1			1			1			1		
		Quartile 2	1·39	1·08, 1·79	0·009	1·15	0·88, 1·49	0·28	1·44	1·11, 1·87	0·005	1·03	0·79, 1·35	0·78
		Quartile 3	2·53	2·00, 3·21	≤0·001	1·96	1·50, 2·55	≤0·001	2·69	2·05, 3·52	0·001	1·62	1·21, 2·17	0·001
		Quartile 4	3·94	3·13, 4·97	≤0·001	2·43	1·81, 3·28	≤0·001	4·25	3·16, 5·70	0·001	1·87	1·33, 2·63	≤0·001
		*P*-trend			≤0·001			≤0·001			0·001			≤0·001
Males	DII	Quartile 1	1			1			1			1		
		Quartile 2	1·58	1·08, 2·29	0·01	1·51	1·04, 2·20	0·03	1·47	1·008, 2·16	0·04	1·48	1·01, 2·18	0·04
		Quartile 3	2·91	2·02, 4·21	≤0·001	2·59	1·76, 3·81	≤0·001	2·51	1·68, 3·75	0·001	2·54	1·69, 3·83	≤0·001
		Quartile 4	3·65	2·57, 5·18	≤0·001	2·96	1·97, 4·43	≤0·001	2·69	1·74, 4·16	0·001	2·74	1·75, 4·31	≤0·001
		*P*-trend			≤0·001			≤0·001						≤0·001
	DIL	Quartile 1	1			1			1			1		
		Quartile 2	1·13	0·80, 1·61	0·47	1·02	0·71, 1·47	0·87	0·94	0·65, 1·36	0·75	0·93	0·65, 1·35	0·73
		Quartile 3	1·56	1·11, 2·21	0·01	1·27	0·87, 1·83	0·20	1·06	0·72, 1·57	0·73	1·05	0·70, 1·56	0·80
		Quartile 4	2·49	1·80, 3·44	≤0·001	1·75	1·18, 2·59	0·005	1·37	0·89, 2·11	0·15	1·33	0·84, 2·10	0·21
		*P*-trend			≤0·001			0·001			0·18			0·13
Females	DII	Quartile 1	1			1			1			1		
		Quartile 2	2·12	1·49, 3·01	≤0·001	1·96	1·36, 2·81	≤0·001	1·77	1·21, 2·59	0·003	1·64	1·11, 2·41	0·01
		Quartile 3	2·17	1·53, 3·08	≤0·001	1·83	1·24, 2·72	0·002	1·55	1·02, 2·37	0·03	1·37	0·88, 2·13	0·15
		Quartile 4	3·88	2·70, 5·56	≤0·001	3·10	2·01, 4·76	≤0·001	2·66	1·67, 4·23	0·001	2·26	1·39, 3·69	0·001
		*P* -trend			≤0·001			≤0·001			0·001			0·005
	DIL	Quartile 1	1			1			1			1		
		Quartile 2	1·28	0·88, 1·87	0·19	1·27	0·85, 1·88	0·23	1·17	0·77, 1·77	0·45	1·11	0·73, 1·69	0·61
		Quartile 3	3·26	2·33, 4·57	≤0·001	3·18	2·15, 4·70	≤0·001	2·83	1·83, 4·36	0·001	2·58	1·65, 4·05	≤0·001
		Quartile 4	3·79	2·63, 5·46	≤0·001	3·71	2·34, 5·89	≤0·001	3·28	1·97, 5·45	0·001	2·90	1·70, 4·93	≤0·001
		*P* -trend			≤0·001			≤0·001			0·001			≤0·001

NAFLD, non-alcoholic fatty liver disease; DII, dietary insulin index; DIL, dietary insulin load.

Model 1: crude analysis.

Model 2: adjusted for age, gender and daily energy intake.

Model 3: additional adjustment for smoking, level of education and physical activity.

Model 4: additional adjustment for BMI.

## Discussion

The present study was carried out to explore the associations of DII and DIL to the odds of NAFLD. The results revealed that adherence to a diet with a high DII was positively related to a greater risk of NAFLD in men and women. Moreover, DIL was directly linked to NAFLD in women, although no significant relationship was found for DIL in men.

In line with the present study, a case–control study by Fatahi *et al.* on 369 adults (200 healthy and 169 NAFLD people)^(24)^, as the only available evidence in this area of research, reported that having a diet with a higher DII is related to increased risk of NAFLD after controlling the potential covariates. The study by Fatahi *et al.* ^(24)^ was limited by its low sample size and lack of subgroup analysis based on the gender of participants. Moreover, the relation of DIL to NAFLD was not investigated in the Fatahi *et al.* ^(24)^ study. In this line, recent studies have identified that higher DII and DIL are the predictors of unfavourable metabolic changes associated with NAFLD, such as metabolic syndrome^(7)^, diabetes^(9)^, obesity^(25)^, increased serum TAG and reduced serum levels of HDL^(14)^. There are some mechanisms explaining the relations of DII and DIL to the odds of NAFLD. Food items with high insulinemic potential are quickly absorbed and transformed into glucose, resulting in a quick elevation in circulating insulin and glucose and then a quick reduction in glucose excursion^(7)^. This rapid reduction in circulating glucose may decrease satiety and trigger hunger, resulting in excessive energy consumption^(7)^, which is linked to greater odds for NAFLD^(26)^. A diet with a high DII and DIL is closely associated with hyperinsulinaemia, which is a potential contributor to inflammation^(27)^, oxidative stress^(28)^ and the accumulation of TAG in hepatocytes^(29)^. DII has been recognised as a risk factor for insulin resistance (IR)^(12)^, resulting in dysregulation in glucose metabolism and increased release of free fatty acids through dysregulated lipolysis that further accentuates the impairment in insulin signalling. Under such metabolic changes, increased circulating glucose due to IR could be converted to fat, which ultimately accumulates in the liver, predisposing people to the development of NAFLD^(24)^. Moreover, IR increases the levels of liver enzymes^(30)^ and increases inflammation^(31)^ and oxidative stress^(31)^, playing a role in the development of NAFLD.

In this study, the significant direct relationship between NAFLD and DIL was identified in females but not in males. The underlying mechanism for this sex difference in the association is unclear; nevertheless, it may be explained by the impact of gonadal hormones on body fat storage, appetite and the metabolism of lipids^(32–34)^. Fat accumulation in the liver could be influenced by gonadal steroids; lower testosterone concentration is related to increased risk of NAFLD in males and lower risk of NAFLD in females^(35)^. While there is no direct evidence showing how testosterone concentration can affect the association between DII and NAFLD risk, diets with high DII may affect serum concentration of testosterone; it has been suggested that higher serum testosterone concentration in women is associated with insulin resistance^(36)^, which is closely associated with NAFLD. Future studies are required to explore the underlying mechanisms for the possible effect of testosterone on the relation of DII to NAFLD risk. Moreover, the difference in circulating adipokines, such as leptin and adiponectin among females and males may be involved in this sex disparity^(7,37,38)^. Evidence has suggested that men and women have different patterns in adipokine secretion due to their differences in fat distribution^(39)^. For example, serum leptin levels are about two times higher in women than in men even when controlling for BMI^(40)^. Adipokines play a key role in the regulation of steatosis and NAFLD^(41)^. Diets with high DIL affect NAFLD through hyperinsulinaemia^(42)^. On the other side, higher concentrations of insulin induce leptin secretion^(43)^. Hyperleptinemia, or persistently high levels of leptin, has been associated with the development of NAFLD^(44)^. Accordingly, higher diets with DIL may result in a greater increase in leptin levels in women than in men, thereby increasing their odds of NAFLD.

In the current study, the mean DII and DIL values were 78·21 ± 19·75 and 784·06 ± 192·35, respectively. In the Nurses’ Health Study on 28 909 from 11 U.S. states, the mean DII (44·40) was lower than the present study, but the mean value for DIL was 702·66, which was comparable with our study^(45)^. The mean of DII and DIL (DII: 51·7 ± 6·5; DIL: 235·8 ± 90·2) of participants in the study by Teymoori *et al.* were relatively lower compared with our study^(13)^. In a study in Afghanistan by Amiry *et al.*, the median DII was 50·75^(46)^; in contrast, the median value of DIL in the population of Afghanistan was 143 792·5^(46)^, which is greater than that seen in the present study. This difference might be due to differences in dietary patterns, type and the number of food items used to calculate DIL, as well as the method used to compute DIL/DII in various studies. Moreover, differences in the type of dietary assessment method may be another reason for differences in the DIL values in various studies.

This is the largest study investigating the link of DII and DIL to NAFLD. The relatively large sample size, the use of a validated FFQ for the assessment of food intake, a sex-stratified analysis and the use of sensitive diagnostic factors such as FLI and fibroscan and confirming the diagnosis of NAFLD by a gastroenterologist are among the strengths of this study. Furthermore, the participants were selected by random sampling of subjects who were referred to nutrition centres, minimising the likelihood of selection bias. Some limitations of the current study should be taken into consideration. First, because of the cross-sectional nature of this study, causality is not inferable, and it is unclear whether DIL and DII change the odds of NAFLD or the disease affects dietary preferences. Thus, prospective investigations are required to confirm our results. Second, despite adjustments for potential covariates, the residual effects of unmeasured/unknown covariates may affect the results. Third, the FFQ is prone to potential reporting biases, such as recall bias, which may influence the findings. Finally, since the DII values for several foods in the FFQ were not available in the database, the DII of similar food items were used for items that were not available in the reference list. Accordingly, additional DII testing is needed to confirm our results.

In conclusion, this study revealed that greater adherence to a diet with a high DII and DIL might be linked to a higher risk of NAFLD in women. In addition, DII was also positively related to increased odds of NAFLD in men. The results of the current study might be useful for healthcare providers to design appropriate preventive measures for people at risk of NAFLD. Additional research, in particular with a prospective cohort design, is required to establish these conclusions.

## Supporting information

Motamedi et al. supplementary material Motamedi et al. supplementary material

## Supplementary material

For supplementary material accompanying this paper visit https://doi.org/10.1017/S1368980024001149
