# Supplementary material for: Dietary insulin index and dietary insulin load in relation to non-alcoholic fatty liver disease: a cross-sectional study
Source: Public Health Nutr. 2024 Sep 26;27(1):e182. doi: 10.1017/S1368980024001149 (PMC11504692; doi:10.1017/S1368980024001149)
Supplement: Motamedi et al. supplementary material [file S1368980024001149sup001.docx]

Table S1. Source of DII and its value for each food item according to the previous studies.

| Row | The source of DII value from previous studies (1-4) | DII^a^ per 1,000 kJ or 239 kcalc |
| --- | --- | --- |
| 1 | Grain bread | 41 ± 4 |
| 2 | White bread | 73 ± 5 |
| 3 | White rice (cooked) | 58 ± 9 |
| 4 | Spiral pasta (cooked) | 29 ± 4 |
| 5 | Whole-meal bread | 70 ± 9 |
| 6 | Beef | 37 ±12 |
| 7 | Chicken, panfried with skin | 19 ± 4 |
| 8 | Lamb | 41 ± 19 |
| 9 | Fish | 43 ± 13 |
| 10 | Egg | 23 ± 4 |
| 11 | Beef sausage | 7 ± 9 |
| 12 | Ham, shaved (Coles) | 19 ± 11 |
| 13 | Pizza | 47 ± 4 |
| 14 | Milk | 24 ± 3 |
| 15 | Yoghurt | 46 ± 19 |
| 16 | Cheese | 33 ± 9 |
| 17 | Chocolate milk | 46 ± 23 |
| 18 | Broccoli | 29 ± 8 |
| 19 | Baked beans | 88 ± 14 |
| 20 | Peas, steamed | 37 ± 8 |
| 21 | Lentils | 42 ± 9 |
| 22 | Tofu | 21 ± 4 |
| 23 | Mixed beans | 34 ± 14 |
| 24 | Broccoli | 29 ± 8 |
| 25 | Coleslaw, commercial | 20 ± 2 |
| 26 | Tomato pasta sauce | 41 ± 8 |
| 27 | Cauliﬂower, steamed | 48 ± 9 |
| 28 | Carrot | 44 ± 7 |
| 29 | Boiled potato | 88 ± 8 |
| 30 | Popcorn | 39 ± 7 |
| 31 | Great grains | 57 ± 7 |
| 32 | All-Bran Complete Wheat Flakes | 55 ± 7 |
| 33 | Melon | 93 ± 15 |
| 34 | Peach, raw | 39 ± 18 |
| 35 | Seedless raisins | 31 ± 5 |
| 36 | Orange | 44 ± 2 |
| 37 | Black grapes | 60 ± 4 |
| 38 | Apple | 43 ± 3 |
| 39 | Banana | 59 ± 4 |
| 40 | Apple juice | 47 ± 2 |
| 41 | Butter | 2 ± 1 |
| 42 | Olive oil | 3 ± 1 |
| 43 | Walnut | 5 ± 1 |
| 44 | Cream | 8 ± 8 |
| 45 | Peanuts, salted and roasted | 15 ± 2 |
| 46 | Raisin Bran cereal | 69 ± 6 |
| 47 | Glucose | 100 |
| 48 | Raspberry jam | 62 ± 9 |
| 49 | Coca-Cola | 44 ± 3 |
| 50 | Ice cream | 65 ± 9 |
| 51 | Cookie | 67 ± 11 |
| 52 | Apple pie | 47 ± 4 |
| 53 | Mars bar | 89 ± 11 |
| 54 | Potato chips | 44 ± 10 |
| 55 | Corn Flakes | 55 ± 6 |
| 56 | Fat-free blueberry mufﬁn | 69 ± 6 |
| 57 | Fruit punch | 76 ± 10 |
| 58 | Plain biscuit | 48 ± 20 |
| 59 | Coleslaw, commercial | 20 ± 2 |

1. DII = Dietary insulin index. The insulin index for 59 food items was obtained from the previous studies published by Sadeghi et al (1), Bao et al (2), Brand-Miller et al (3) and Bell et al (4).

References:

1. Sadeghi O, Hasani H, Mozaffari-Khosravi H, Maleki V, Lotfi MH, Mirzaei M. Dietary insulin index and dietary insulin load in relation to metabolic syndrome: the Shahedieh cohort study. Journal of the Academy of Nutrition and Dietetics. 2020;120(10):1672-86. e4.

2. Bao J, Atkinson F, Petocz P, Willett WC, Brand-Miller JC. Prediction of postprandial glycemia and insulinemia in lean, young, healthy adults: glycemic load compared with carbohydrate content alone. The American journal of clinical nutrition. 2011;93(5):984-96.

3. Holt S, Miller J, Petocz P. An insulin index of foods: the insulin demand generated by 1000-kJ portions of common foods. The American journal of clinical nutrition. 1997;66(5):1264-76.

4. Bell KJ, Petocz P, Colagiuri S, Brand-Miller JC. Algorithms to improve the prediction of postprandial insulinaemia in response to common foods. Nutrients. 2016;8(4):210.

Table S2. Dietary intake of participants across quartiles of dietary insulin index/load

|  | Quartiles of Dietary insulin index | | | |  | Quartiles of Dietary insulin load | | | |  |
| --- | --- | --- | --- | --- | --- | --- | --- | --- | --- | --- |
|  |  | | | |  |  | | | |  |
|  | Quartile 1 | Quartile 2 | Quartile 3 | Quartile 4 | p-value | Quartile 1 | Quartile 2 | Quartile 3 | Quartile 4 | p-value |
| Energy (Kcal/d  ) | 2309.69  ±400.66 | 2360.82  ±428.56 | 2454.14  ±449.18 | 2616.63  ±447.96 | ≤0.001 | 2351.15  ±420.78 | 2416.67  ±445.83 | 2401.25  ±420.36 | 2572.21  ±470.51 | ≤0.001 |
| Whole grains (gr/d) | 67.34 ±8.91 | 52.37 ±9.25 | 48.48 ±8.79 | 41.79 ±9.20 | ≤0.001 | 64.62 ±10.06 | 55.99±10.78 | 48.51±8.72 | 40.86±8.59 | ≤0.001 |
| Refined grains (gr/d) | 478.14  ±83.95 | 485.81  ±120.58 | 546.98  ±135.02 | 646.38  ±86.38 | ≤0.001 | 521.01  ±108.98 | 528.03  ±127 | 495.60  ±129.52 | 612.68  ±113.50 | ≤0.001 |
| Legumes (gr/d) | 38.66  ±6.28 | 31.49  ±5.57 | 29.95  ±4.9 | 27.42  ±4.41 | ≤0.001 | 37.31  ±6.7 | 33.26  ±6.34 | 29.69  ±4.84 | 27.26  ±4.2 | ≤0.001 |
| Processed meat (gr/d) | 3.1±  0.94 | 2.7  ±1.02 | 2.78  ±0.89 | 2.99  ±1.2 | ≤0.001 | 3.21  ±1.13 | 3.18  ±1.23 | 2.52  ±0.9 | 2.66  ±0.53 | ≤0.001 |
| Nuts (gr/d) | 65.64  ±6.33 | 53.29  ±6.92 | 50.91  ±6.36 | 46.24  ±7.31 | ≤0.001 | 63.74  ±6.71 | 56.71  ±8.06 | 50.46  ±6.57 | 45.18  ±6.47 | ≤0.001 |
| Dairies (gr/d) | 360.11  ±45.2 | 289.63  ±34.95 | 267.57  ±32.7 | 236.72  ±30.25 | ≤0.001 | 339.14  ±56.4 | 299.45  ±48.81 | 275.8  ±36.78 | 239.61  ±36.72 | ≤0.001 |
| Vegetables (gr/d) | 361.59  ±40.84 | 330.08  ±43.1 | 308.76  ±45.44 | 264.74  ±36.84 | ≤0.001 | 347.05  ±43.76 | 329.68  ±42.96 | 320.37  ±50.24 | 268.05  ±46.39 | ≤0.001 |
| Fruits  (gr/d) | 437.55  ±29.02 | 388.88  ±30.48 | 368.88  ±34.39 | 334.82  ±36.66 | ≤0.001 | 421.68  ±41.03 | 393.73  ±39.65 | 376  ±34.51 | 337.84  ±40.93 | ≤0.001 |
| Red meat  (gr/d) | 38.79  ±6.24 | 32.1  ±4.95 | 30.63  ±4.83 | 27.77  ±4.94 | ≤0.001 | 37.92  ±5.84 | 34.29  ±5.91 | 30.26  ±4.63 | 26.81  ±4.02 | ≤0.001 |
| soft drinks (gr/d) | 55.12 ± 5.21 | 56.01 ± 8.11 | 66.53 ± 7.45 | 79.58±10.87 | ≤0.001 | 54.36±4.96 | 57.32±3.25 | 61.65± 0.85 | 83.91±13.64 | ≤0.001 |
| Sweets/confectionary (gr/d) | 82.35 ±15.97 | 88.34±13.25 | 93.41±16.58 | 96.36±14.65 | ≤0.001 | 84.93±19.36 | 89.46±21.25 | 85.62± 17.93 | 100.45±21.38 | ≤0.001 |

Table S3. Pearson's correlation coefficient between dietary groups and dietary insulin index/load after adjustment for sex and age

|  | Dietary insulin index | | Dietary insulin load | |
| --- | --- | --- | --- | --- |
|  | r | p-value | r | p-value |
| Energy | 0.16 | ≤0.001 | 0.08 | ≤0.001 |
| Whole grains | -0.42 | ≤0.001 | -0.20 | ≤0.001 |
| Refined grains | 0.51 | ≤0.001 | 0.26 | ≤0.001 |
| Legumes | -0.28 | ≤0.001 | -0.12 | ≤0.001 |
| Processed meat | -0.46 | ≤0.001 | -0.19 | ≤0.001 |
| Nuts | -0.40 | ≤0.001 | -0.18 | ≤0.001 |
| Dairies | -0.51 | ≤0.001 | -0.23 | ≤0.001 |
| Vegetables | -0.43 | ≤0.001 | -0.23 | ≤0.001 |
| Fruits | -0.52 | ≤0.001 | -0.23 | ≤0.001 |
| Red meat | -0.25 | ≤0.001 | -0.10 | ≤0.001 |
| soft drinks | 0.41 | ≤0.001 | 0.16 | ≤0.001 |
| Sweets/confectionary | 0.44 | ≤0.001 | 0.18 | ≤0.001 |

r: Pearson's correlation coefficient
